# Comparison of Clinical Effects of Essential Oil–Containing Mouthwash and Chlorhexidine Mouth Wash in Patients Undergoing Fixed Orthodontic Treatment – Three‐Month Follow‐up Randomized‐Controlled Trial

**DOI:** 10.1002/cre2.70477

**Published:** 2026-10-06

**Authors:** Fazli Rabi, Naveed Sadiq, Muhammad Jamil, Sadia Asif Samdani, Waqas Naseem, Ferdous Bukhary, Umar Hussain, Alessandra Campobasso, Nikolaos Pandis

**Affiliations:** ^1^ Department of Public Health MS Epidemiology and Biostatistics Scholar Khyber Medical University Peshawar Khyber Pakhtunkhwa Pakistan; ^2^ Institute of Public Health and Social Sciences Khyber Medical University Peshawar Khyber Pakhtunkhwa Pakistan; ^3^ Periodontology, Khyber College of Dentistry Peshawar Khyber Pakhtunkhwa Pakistan; ^4^ Department of Orthodontics Isra University Hyderabad Sindh Pakistan; ^5^ Department of Health Sciences University of Debrecen Debrecen Hajdú‐Bihar Hungary; ^6^ Department of Preventive Dental Sciences, College of Dentistry Dar Al Uloom University Riyadh Saudi Arabia; ^7^ Orthodontics, Saidu College of Dentistry Swat Khyber Pakhtunkhwa Pakistan; ^8^ Orthodontics, Melbourne Dental School, Faculty of Medicine, Dentistry and Health Sciences University of Melbourne Melbourne Victoria Australia; ^9^ University of Bern Bern Switzerland

**Keywords:** chlorhexidine, gingival index, listerine, orthodontics, plaque index

## Abstract

**Objective:**

This study aimed to compare the clinical effects of chlorhexidine (CHX) and essential oil–containing mouthwash (EOM) on periodontal health in patients undergoing fixed orthodontic treatment over a 3‐month follow‐up period.

**Material and Methods:**

This double‐blinded, parallel‐arm randomized‐controlled clinical trial with a 1:1 allocation ratio was conducted at the Department of Orthodontics, Saidu College of Dentistry, Swat, Pakistan. Sixty participants aged 13–40 years undergoing fixed orthodontic treatment were randomly allocated to receive either 0.12% chlorhexidine mouthwash or Listerine essential oil mouthwash, dispensed in identical coded containers to ensure blinding. Participants were instructed to rinse with 10 mL of the assigned mouthwash twice daily for 30 s for 3 months while maintaining routine oral hygiene practices. The primary outcomes were the plaque index and the gingival index. Tooth staining was assessed as a secondary outcome (harm). Clinical outcomes, including the plaque index, the gingival index, and the stain index, were recorded at baseline, 1, 2, and 3 months by a calibrated examiner. Data were analyzed using generalized estimating equation models to evaluate longitudinal changes between groups, with statistical significance set at *p* ≤ 0.05.

**Results:**

The gingival index showed a significant change over time (Wald *p* = 0.02), with no statistically significant overall difference between the CHX and Listerine groups (Wald *p* = 0.07). The plaque index was significantly higher in the CHX group than in the Listerine group (Wald *p* < 0.001). The stain index was also significantly higher in the CHX group than in the Listerine group (Wald *p* < 0.001).

**Conclusion:**

Both mouthwashes were associated with changes in periodontal health during fixed orthodontic treatment. Compared with CHX, EOM was associated with significantly lower plaque index and stain index scores, whereas no statistically significant overall difference between groups was observed for the gingival index.

**Trial Registration:** This trial was retrospectively registered at ClinicalTrials.gov (Identifier: NCT07119840).

## 1 Introduction

### 1.1 Background

Orthodontic treatment with fixed mechanotherapy can substantially alter the composition and balance of the normal oral microbiota (Kado et al. 2020). Inadequate oral hygiene may lead to the accumulation of dental biofilm around orthodontic brackets and bands, subsequently resulting in gingival inflammation and edema (Zhao et al. 2024). Effective mechanical plaque control is therefore recommended to prevent the onset of gingivitis, periodontitis, and dental caries during fixed appliance treatment (Di Spirito et al. 2023). However, maintaining optimal oral hygiene during fixed orthodontic treatment is often challenging due to the presence of wires, ligatures, bands, and other appliance components that create additional plaque‐retentive sites (Hussain et al. 2023).

In addition to oral hygiene instruction, the use of chemotherapeutic anti‐plaque agents can substantially reduce the bacterial load in the oral cavity (Brookes et al. 2021). Chlorhexidine (CHX) is the most widely used agent and is regarded as the gold standard due to its broad‐spectrum antimicrobial activity, low toxicity, and high substantivity to epithelial tissues and oral mucosa (Hussain et al. 2023; Alavi and Yaraghi 2018). CHX can reduce salivary microbial counts by up to 90% (Raju et al. 2017).

An antiseptic mouthrinse containing essential oils (Listerine) has been used in clinical practice for many decades. Miller first referred to it in 1890 as a highly effective antiseptic, and a subsequent independent evaluation published in *The Lancet* in 1929 confirmed its wide antibacterial effects and safety profile. Its active components include a standardized combination of essential oils: thymol (0.064%), eucalyptol (0.092%), methyl salicylate (0.060%), and menthol (0.042%) (Zaitouna et al. 2017). Long‐term clinical trials conducted in accordance with standardized evaluation guidelines have further confirmed its antiplaque and antigingivitis efficacy when used as an adjunct to routine oral hygiene over 6 months, without promoting calculus formation or extrinsic staining (Sharma et al. 2004; Zaitouna et al. 2017).

### 1.2 Rationale and Literature Gap

Although studies have compared chlorhexidine (CHX) mouthwash with essential oil–containing mouthwash (EOM) in the general population, limited evidence is available regarding their comparative clinical effects in patients undergoing fixed orthodontic treatment (Zaitouna et al. 2017; Charles et al. 2004; Singh et al. 2013; Quintas et al. 2015). To our knowledge, only one study has compared CHX with EOM in orthodontic patients; however, it has notable limitations. The sample size was small (*n* = 30), reducing statistical power, and the follow‐up period was only 15 days, which is too short to assess sustained effects (Raju et al. 2017). The randomization procedure was not clearly described, and true blinding is questionable due to the distinct taste and characteristics of the mouthwashes. Although a cross‐over design, three‐arm trial was used, the 15‐day washout period is insufficient to counter the prolonged substantivity of CHX, creating a risk of carry‐over bias. This problem was compounded by the fact that only two groups were crossed over, whereas the CHX group remained unchanged, resulting in an asymmetric and less reliable comparison.

Fixed appliances predispose orthodontic patients to plaque accumulation and gingival inflammation, so there is a need for safe and effective chemical adjuncts. Although CHX remains an effective agent for short‐term antiplaque control (Hussain et al. 2023), its adverse effects (staining, taste alteration, and mucosal irritation) limit sustained use (Yaneva et al. 2022; Kouadio et al. 2017). Essential oil mouthrinses such as EOM offer better long‐term tolerability with moderate efficacy (Goes et al. 2016). However, conventional alcohol‐containing formulations may cause oral burning or mucosal irritation in some individuals and may reduce patient acceptance during prolonged use. Despite these considerations, comparative evidence in orthodontic populations is lacking. A well‐designed randomized‐controlled trial is therefore required to determine whether EOM can provide clinically acceptable outcomes with fewer side effects than CHX in patients undergoing fixed orthodontic treatment.

### 1.3 Specific Objectives and Hypotheses

The specific objective of this study was to compare the clinical effects of CHX and essential oil–containing mouthrinse on periodontal health—including plaque accumulation, gingival inflammation, and dental staining—in patients undergoing fixed orthodontic treatment over a 3‐month follow‐up period.

The study hypothesized that an essential oil‐containing mouthrinse would result in different periodontal outcomes compared with a chlorhexidine‐containing mouthrinse and would be associated with less dental staining during fixed orthodontic treatment.

## 2 Materials and Methods

### 2.1 Trial Design

This was a double‐blinded, parallel‐arm, superiority randomized‐ controlled clinical trial with a 1:1 allocation ratio. The reporting of this randomized clinical trial adheres to the Consolidated Standards of Reporting Trials (CONSORT) guidelines. Patients and members of the public were not involved in the design, conduct, reporting, or dissemination plans of this trial.

### 2.2 Trial Registration

The study was prospectively conducted as a randomized‐controlled trial; however, the trial was retrospectively registered at ClinicalTrials.gov (Identifier: NCT07119840). The registered protocol specified outcome assessments at baseline, 4 weeks, and 8 weeks. A 3‐month assessment was subsequently added as an extension of the follow‐up period to provide information on the longer‐term persistence of the effects of EOM and CHX on gingival and plaque indices in patients undergoing fixed orthodontic treatment. This constituted a deviation from the registered follow‐up schedule. The same clinical indices and examination procedures were maintained, and there were no changes to the study design, allocation, intervention, primary outcomes, or statistical analysis. The study protocol and statistical analysis plan were not published before trial commencement, but are available from the corresponding author upon reasonable request.

### 2.3 Ethical Approval and Informed Consent

Ethical approval was obtained from the Departmental Review Board (Ref No.: SCD/Ortho/EC/021/2025), Department of Orthodontics, Saidu College of Dentistry, Swat, Pakistan, prior to the commencement of the study. Written informed consent was obtained from all participants; for participants younger than 16 years of age, consent was obtained from a parent or legal guardian, and verbal assent was also obtained from the participant. All procedures were conducted in accordance with the Declaration of Helsinki for ethical standards in human research.

### 2.4 Participants, Eligibility Criteria, and Settings

Individuals between 13 and 40 years of age with a full permanent dentition, including fully erupted first molars bonded with molar tubes, no clinical signs of periodontitis, and who were willing to undergo conventional metallic fixed orthodontic treatment and comply with the recommended oral hygiene instructions were included in the study. Baseline clinical assessments were performed immediately before bonding of the fixed orthodontic appliances and prior to commencement of the allocated mouthwash intervention. Patients with any systemic condition known to affect gingival or periodontal health, including diabetes, hypertension, asthma, hepatitis, leukemia, cardiac disease, or known allergies, as well as those with heavily restored labial tooth surfaces, were excluded. Patients who had received systemic antibiotics, anti‐inflammatory medications, or professional periodontal therapy within the preceding 3 months were also excluded. Pregnant or lactating women, individuals with a history of tobacco use, those reporting allergies to CHX or Listerine components, patients who had used any mouthwash continuously during the preceding 3 months, and patients demonstrating abnormal parafunctional oral habits were also excluded. Participants with active untreated dental caries requiring immediate operative treatment were not enrolled.

This study was conducted at the Department of Orthodontics, Saidu College of Dentistry, Swat. Participant recruitment was undertaken from May 2, 2025 to June 20, 2025 using a non‐probability consecutive sampling technique. Following baseline assessment and randomization, each participant commenced the allocated intervention on the day of enrollment. Clinical outcome assessments were performed at baseline and after 1, 2, and 3 months. Follow‐up of the last enrolled participant was completed on September 20, 2025, marking the end of the trial.

### 2.5 Interventions

As part of the routine departmental protocol, all participants underwent professional periodontal prophylaxis before baseline assessment to standardize oral hygiene conditions prior to the initiation of orthodontic treatment. Following baseline assessment, participants were randomly allocated to one of the two study groups. Both mouthwashes—0.12% Chlorhexidine gluconate mouthwash (Clinical, manufactured by Platinum Pharmaceuticals Pvt. Ltd.) and Listerine essential oil–based mouthwash (manufactured by Parke Davis & Co. Ltd.)—were purchased from the market and dispensed in identical, opaque, coded containers to ensure blinding. The EOM used in this study was an alcohol‐free formulation containing zinc chloride (0.09%), eucalyptol (0.092%), thymol (0.064%), methyl salicylate (0.060%), and menthol (0.042%). All labeling and brand information was concealed from both the participants and the investigator recording the clinical indices (Figure 1). Group A received a commercially available mouthwash containing 0.12% chlorhexidine gluconate (Clinical, Platinum Pharmaceuticals Pvt. Ltd.) and Group B received EOM (Listerine), which was an alcohol‐free formulation containing eucalyptol (0.092%), menthol (0.042%), methyl salicylate (0.060%), and thymol (0.064%). The product was manufactured by Parke Davis & Co. Ltd.

**Figure 1:**
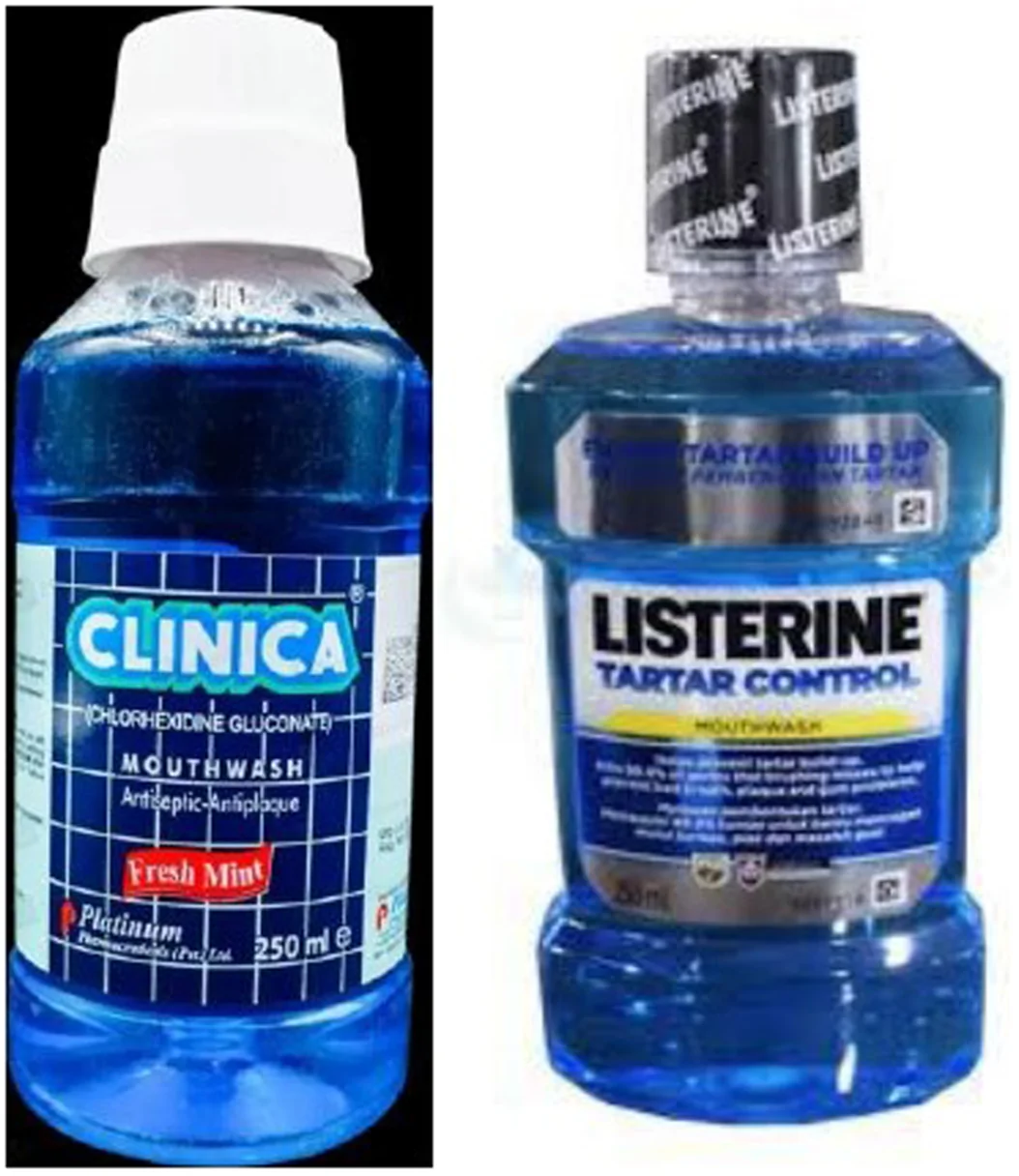
Mouthwashes purchased from the market with similar colors.

Participants in both groups were instructed to maintain routine oral hygiene practices, including brushing twice daily with fluoride toothpaste. The assigned mouthwash was to be used twice daily, once in the morning and once at night, following regular brushing. Each participant was instructed to rinse with 10 mL of the allocated mouthwash for 30 s, without rinsing with water afterward, and to refrain from eating or drinking for 30 min following its use. No specific waiting interval between toothbrushing and mouthwash use was prescribed. Standardized oral hygiene instructions were delivered verbally and reinforced through written guidelines at the baseline visit and at each subsequent follow‐up.

Compliance was monitored at monthly visits by patient self‐reporting and by visually assessing the volume of the remaining mouthwash in the issued containers. Returned mouthwash volumes were not measured quantitatively, missed doses were not formally recorded, and no predefined adherence threshold was specified. Participants were advised not to use any additional mouthrinses, interdental cleaning aids, medicated gels, or other adjunctive oral hygiene products during the study. Clinical examinations and outcome measurements (plaque index, gingival index, and stain score) were recorded at baseline, 1 month, 2 months, and 3 months.

### 2.6 Outcomes

The primary outcomes were plaque index and gingival index, assessed longitudinally from baseline (before bonding) to the 3‐month follow‐up. The principal comparison was based on the overall differences in these outcomes between treatment groups over time using generalized estimating equation (GEE) models. The tooth stain index was prespecified as a secondary outcome (harm outcome).

The plaque index was assessed using a modified plaque scoring system based on the principles of the Silness and Löe plaque index to evaluate plaque accumulation around orthodontic brackets. The plaque index was assessed by examining plaque on each aspect of the bracket base, including the mesial, distal, occlusal, and gingival surfaces of all bonded teeth. Grade 0 indicated no plaque around the bracket base, Grade 1 indicated plaque deposits on one surface, Grade 2 indicated plaque deposits on two surfaces, Grade 3 indicated plaque deposits on three surfaces, and Grade 4 indicated plaque deposits on all four surfaces and/or the presence of gingival inflammation. A score of 0 represented the absence of plaque and inflammation, whereas scores 1–3 reflected increasing severity of plaque deposits, and score 4 also incorporated signs of gingival inflammation. The highest score for each tooth was recorded based on all four aspects, then averaged per tooth, and subsequently, the mean score was calculated across 20 teeth (from the first permanent molar on one side to its contralateral counterpart in both jaws). This assessment was performed at baseline and again at 1, 2, and 3 months.

Gingival inflammation was assessed using the Löe and Silness gingival index. The gingival index was assessed by grading the gingival condition on each tooth's labial surface as follows: Grade 0 represented normal gingiva, Grade 1 indicated mild inflammation with slight color change and no bleeding on probing, Grade 2 indicated moderate inflammation with redness, edema, glazing, or bleeding on probing, and Grade 3 indicated severe inflammation with marked redness, edema, and a tendency for spontaneous bleeding. The individual gingival index was calculated by dividing the total score by the number of examined teeth (*n* = 20).

The stain score was recorded using a simplified stain area index. The labial surfaces of the 20 scorable teeth (excluding any fully banded or extensively restored teeth) were examined. For teeth with bonded brackets, only the visible enamel around the bracket was assessed. After gentle drying, each tooth surface was assigned a single stain area score based on the amount of visible enamel covered by stain: 0 indicated no stain, 1 indicated stain covering up to one‐third of the surface, 2 indicated stain covering more than one‐third but up to two‐thirds of the surface, and 3 indicated stain covering more than two‐thirds of the surface. The stain scores of all examined teeth were summed and divided by the number of teeth scored to obtain the mean stain score for each subject. This assessment was also conducted at baseline, and after 1, 2, and 3 months.

A single investigator (FR) performed data collection. Five orthodontic patients (providing 20 scorable teeth each) representing a range of plaque, gingival, and stain conditions were examined. The examiner recorded PI, GI, and stain index scores for these patients, and the assessments were repeated after 10 days under similar clinical conditions while remaining blinded to the initial recordings. Intra‐examiner reliability was assessed using weighted Cohen's kappa (quadratic weights) for tooth‐level ordinal scores and the intraclass correlation coefficient (ICC; two‐way random‐effects model, absolute agreement) for averaged subject‐level scores.

### 2.7 Sample Size Calculation

The initial sample size was calculated based on the expected difference in the plaque index between the chlorhexidine (CHX) and essential oil mouthwash groups reported by Raju et al. For a two‐group comparison of means, the sample size was estimated using the formula *n* = 2(Z_1−_α/_2_ + Z_1−_β)^2^σ_p_^2^/Δ^2^, where Δ is the expected mean difference and *σ*_p_ is the pooled standard deviation. The reported mean plaque index values were 2.08 (SD = 0.30) for the CHX group and 1.48 (SD = 0.47) for the essential oil mouthwash group, yielding Δ = 0.60. With *α* = 0.05% and 90% power, the minimum required sample size was 20 participants (10 per group). As the primary analysis used GEEs with four repeated measurements and an assumed within‐participant correlation of *ρ* = 0.30, the correlation‐related design effect was calculated as DE = 1 + (*m* − 1)*ρ* = 1 + (4 − 1)(0.30) = 1.90. For the achieved total sample of 60 participants, the correlation‐adjusted effective sample size was N_eff = N/DE = 60/1.90 = 31.58, supporting the adequacy of the achieved sample for the longitudinal GEE analysis. The final study included 60 participants, with 30 participants in each group, and all participants completed the scheduled follow‐up assessments.

### 2.8 Safety Monitoring

Participant safety and treatment tolerance were monitored at the 1‐month follow‐up by the principal investigator. Descriptive comparisons of plaque index and gingival index scores were reviewed, together with observation for adverse reactions such as oral irritation, altered taste, burning sensation, or allergic responses to the assigned mouthwashes. No formal interim efficacy analysis or statistical testing was performed. As both interventions included commercially available mouthwashes with established safety profiles, no prespecified stopping rules were defined. No safety concerns were identified, and the study continued as planned.

### 2.9 Randomization (Random Number Generation, Allocation Concealment, and Implementation)

Patients attending the Orthodontics outpatient department were screened for eligibility. A detailed medical and dental history was recorded, followed by a thorough clinical oral examination conducted in a well‐illuminated operatory environment. Age and standard oral hygiene instructions (twice‐daily brushing with a fluoride toothpaste and avoidance of additional mouthwash use outside the study protocol) were demonstrated to all potential participants to ensure a uniform baseline oral hygiene routine. Participants who fulfilled the inclusion criteria and had none of the exclusion criteria were enrolled in the study. Allocation into Group A (Chlorhexidine) or Group B (essential oil–containing mouthwash) was carried out using a computer‐generated randomization list (RAND function in Microsoft Excel) to ensure equal probability of assignment. Allocation concealment was maintained by placing group assignments into sequentially numbered, opaque, sealed envelopes, opened only at the time of dispensing the mouthwash. Both types of mouthwashes were provided in coded containers with brand and product identifiers concealed to maintain blinding of the outcome assessor.

### 2.10 Blinding

A double‐blind design was used to minimize bias. Both the participants and the outcome assessor were blinded to group allocation. The mouthwashes (0.12% chlorhexidine gluconate and Listerine essential oil–based) were dispensed in their original manufacturer bottles, with all labels and brand identifiers concealed by a sealed paper covering before dispensing to participants. The bottles were of similar colors, although their original shapes differed slightly. Participants were not shown the alternative mouthwash and were instructed not to disclose their allocated mouthwash during follow‐up visits. To ensure that the outcome assessor remained blinded, all clinical examinations were conducted without the assessor viewing the bottles, and participants were instructed not to reveal which mouthwash they had received. The assessor recorded plaque index, gingival index, and stain scores using coded participant IDs only, with allocation codes kept separate and only revealed after completion of data collection. Because the two mouthwashes differed in taste and aroma, complete participant blinding could not be guaranteed, and formal assessment of blinding success was not performed.

### 2.11 Statistical Analysis

Data were analyzed using R (version 4.2) and Stata New 19.5 (Stata Corp, TX, USA). Descriptive statistics were calculated for all variables. Frequencies and percentages were reported for categorical variables (e.g., gender), whereas means and standard deviations were calculated for quantitative variables, including age, PI, GI, and stain. To evaluate changes in PI, GI, and stain index scores over time between the two intervention groups, population‐averaged GEE models with robust standard errors and an exchangeable correlation structure were fitted to assess the effect of treatment, time, and their interaction on the outcomes. Age and gender were considered a priori confounders and were included as covariates in the GEE models. The Wald test was used to statistically assess the main effects (treatment, time) and their interaction treatment × time and marginal plots were drafted to better visualize the effects. Model adequacy was assessed by examining residual plots, observed versus predicted marginal values, and participant‐level trajectory plots for evidence of systematic departures from model assumptions or influential observations. Agreement was assessed using the kappa statistic or the ICC depending on the outcome. Statistical significance was set at *p* ≤ 0.05.

## 3 Results

### 3.1 Participant Flow

Eighty participants were assessed for eligibility; 20 were excluded (18 did not meet the inclusion criteria and 2 declined to participate). Sixty participants were randomized in a 1:1 ratio to either Listerine mouthwash (*n* = 30) or chlorhexidine (CHX) mouthwash (*n* = 30). All allocated participants received the assigned intervention, with no losses to follow‐up or discontinuations in either group. Consequently, all 60 randomized participants were included in the final analysis (Figure 2).

**Figure 2:**
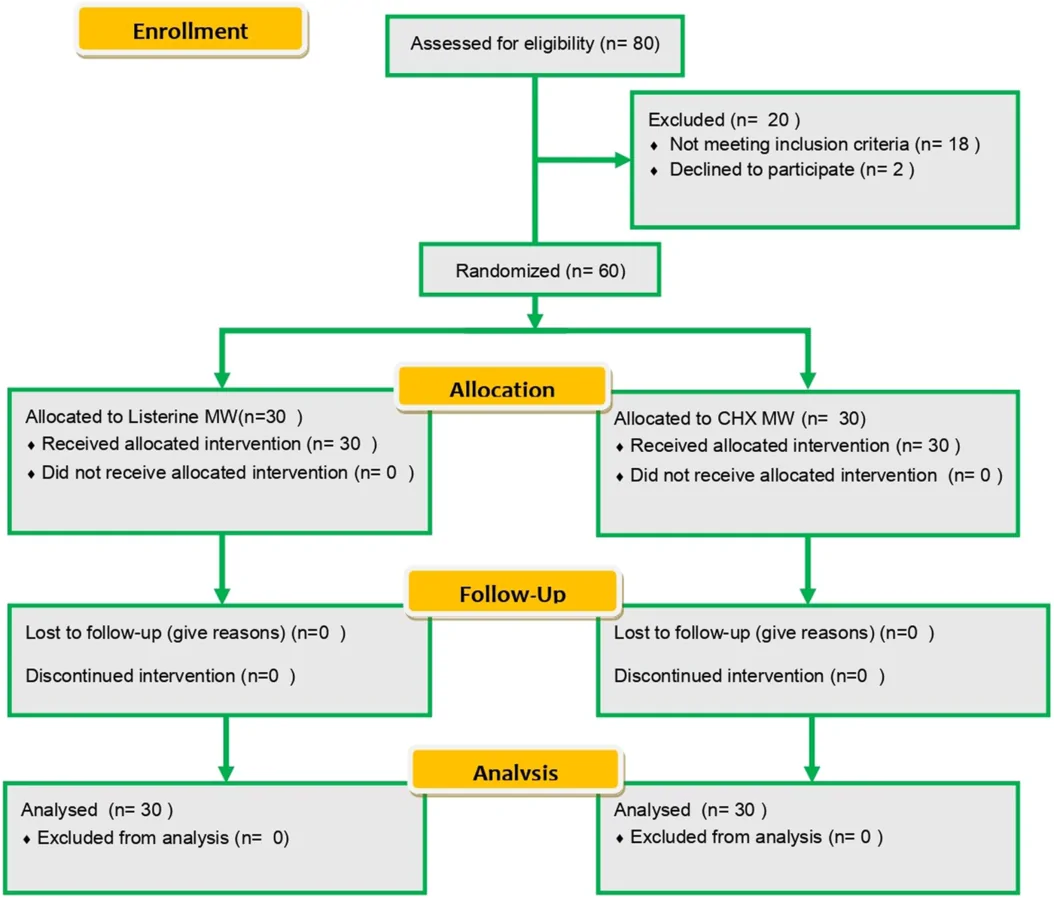
CONSORT flowchart of participants through the trial.

### 3.2 Model Diagnostics

Diagnostic plots showed randomly distributed residuals without systematic trends and good agreement between observed and predicted values. No evidence of influential observations or model misspecification was identified, supporting the adequacy of the fitted GEE models.

### 3.3 Intra‐Examiner Reliability

Reliability was acceptable. The intra‐examiner reliability was acceptable, with a weighted Cohen's kappa of 0.78 and an ICC of 0.82.

### 3.4 Baseline Data

A total of 60 participants were included, with 30 in each group. The gender distribution was similar between groups, with females comprising 57% of the CHX group and 60% of the Listerine group. The median age was 25.5 years (IQR 19.0–34.0) in the CHX group and 30.0 years (IQR 24.0–37.0) in the Listerine group. Baseline gingival and plaque indices were similar between groups (Table 1).

**Table 1:** Baseline demographic and clinical characteristics of participants in the CHX and EOM groups.

Characteristic	CHX mouthwash *n* = 30	EO mouthwash *n* = 30
Gender, *n* (%)		
Female	17 (56.67)	18 (60.00)
Male	13 (43.33)	12 (40.00)
Age in years, median (IQR)	25.50 (19.0, 34.0)	30.00 (24.0, 37.0)
Baseline gingival index, mean (SD)	1.21 (0.03)	1.28 (0.26)
Baseline plaque index, mean (SD)	1.31 (0.04)	1.38 (0.06)
Baseline stain index, mean (SD)	0.43 (0.57)	0.47 (0.63)

### 3.5 Descriptive Outcomes Over Time

The mean gingival index, plaque index, and stain index values for both groups at baseline and each follow‐up visit are presented in Table 2. At baseline, all three outcomes were comparable between the CHX and Listerine groups. During follow‐up, the Listerine group consistently demonstrated lower mean plaque index and stain index values than the CHX group, whereas differences in the gingival index remained relatively small throughout the study period.

**Table 2:** Mean (SD) values of the gingival index, the plaque index, and the stain index at baseline and during follow‐up in chlorhexidine (CHX) and EOM groups, with between‐group mean differences.

Time	Variable	CHX (*n* = 30) Mean (SD)	EOM (*n* = 30) Mean (SD)	Mean difference (CHX − listerine) (95% CI)
Baseline	GI	1.21 (0.03)	1.28 (0.26)	−0.07 (− 0.17 to 0.03)
	PI	1.31 (0.04)	1.32 (0.06)	−0.01 (− 0.03 to 0.02)
	Stain	0.43 (0.57)	0.47 (0.63)	−0.03 (− 0.34 to 0.28)
1 month	GI	1.46 (0.55)	1.33 (0.51)	0.13 (− 0.14 to 0.41)
	PI	2.05 (0.26)	1.61 (0.45)	0.44 (0.25 to 0.63)
	Stain	1.20 (0.72)	0.55 (0.39)	0.64 (0.34 to 0.94)
2 months	GI	1.40 (0.48)	1.25 (0.46)	0.15 (− 0.09 to 0.39)
	PI	1.85 (0.25)	1.41 (0.45)	0.44 (0.25 to 0.63)
	Stain	1.41 (0.84)	0.98 (0.59)	0.43 (0.06 to 0.81)
3 months	GI	1.27 (0.43)	1.00 (0.45)	0.27 (0.04 to 0.50)
	PI	1.63 (0.26)	1.21 (0.46)	0.42 (0.22 to 0.61)
	Stain	1.81 (0.80)	1.08 (0.74)	0.73 (0.33 to 1.13)

*Note:* Values are presented as mean (SD).

Abbreviations: GI = gingival index, PI = plaque index.

Spaghetti plots (Figures 3, 4, 5) show the evolution of the outcomes over time and treatment arm. For the stain index, the average values over time and individual participant trajectories with median splines are additionally presented in Figure S1. For all outcomes, considerable variability was evident among participants and over time.

**Figure 3:**
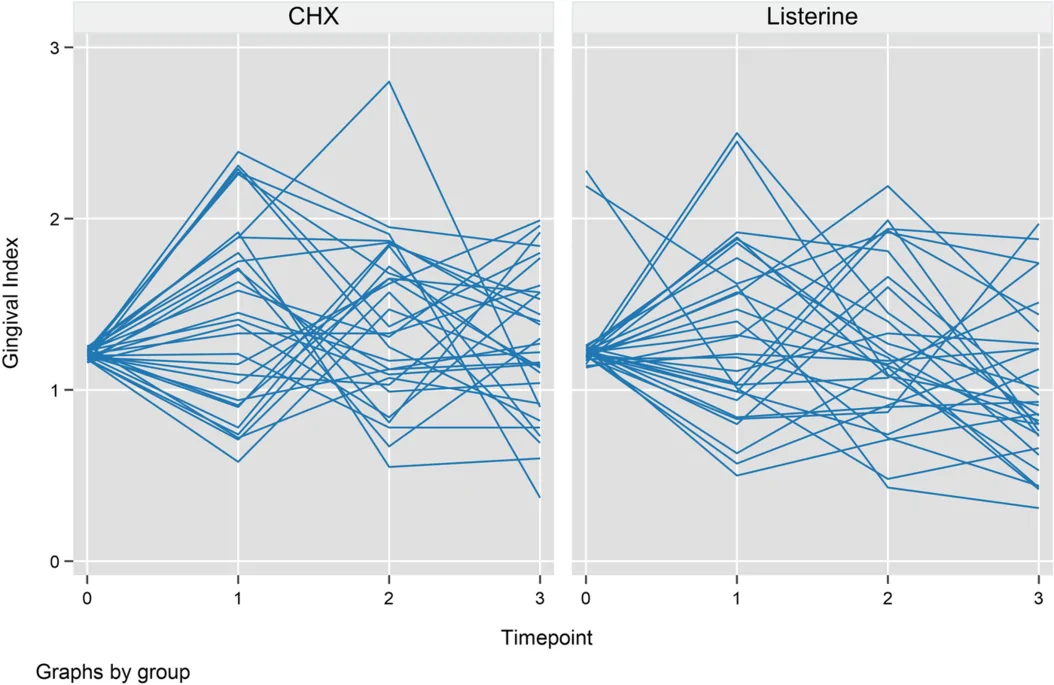
Individual line plots per time point by treatment arm for the gingival index.

**Figure 4:**
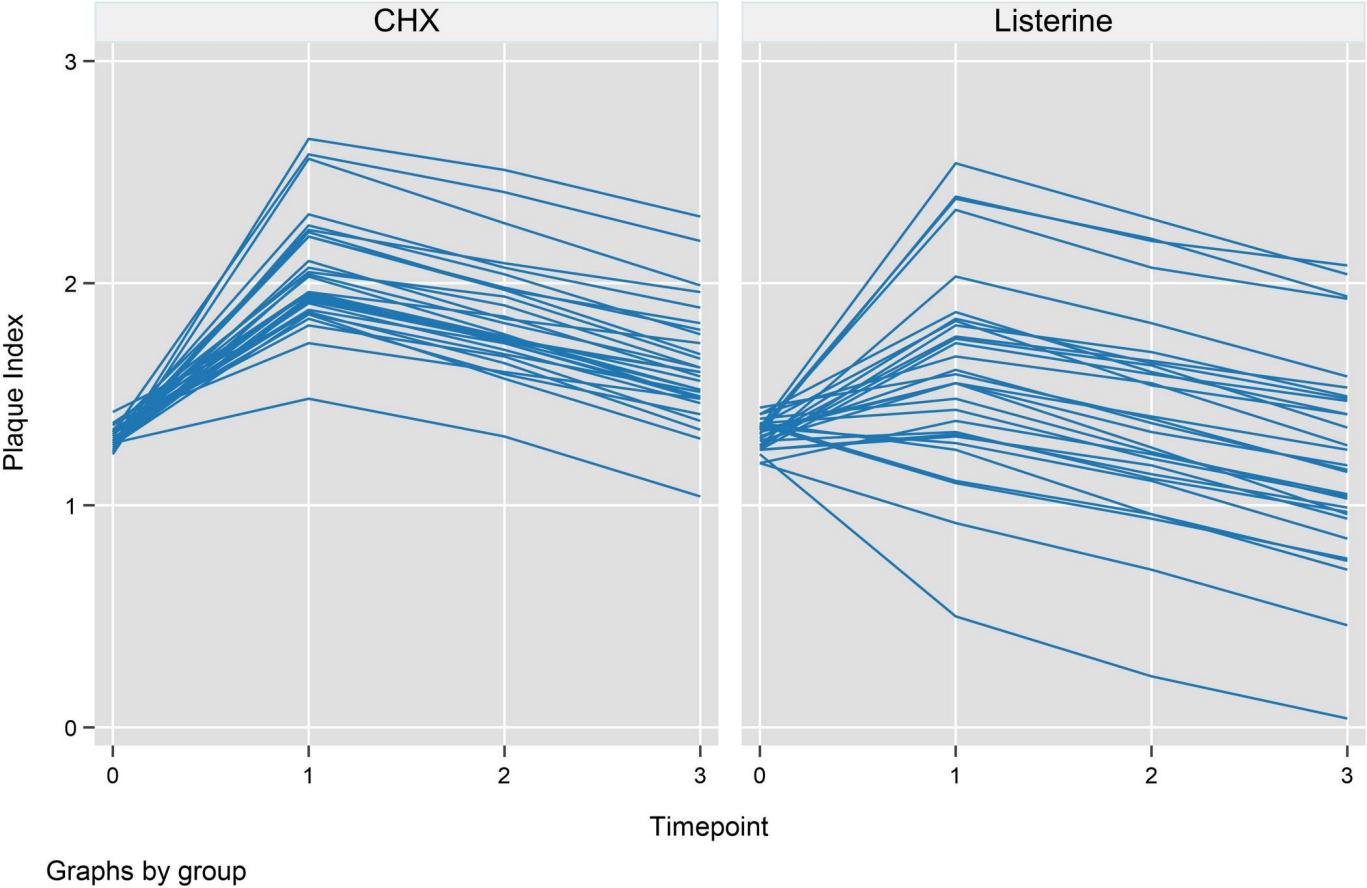
Individual plots per time point per treatment arm for the plaque index.

**Figure 5:**
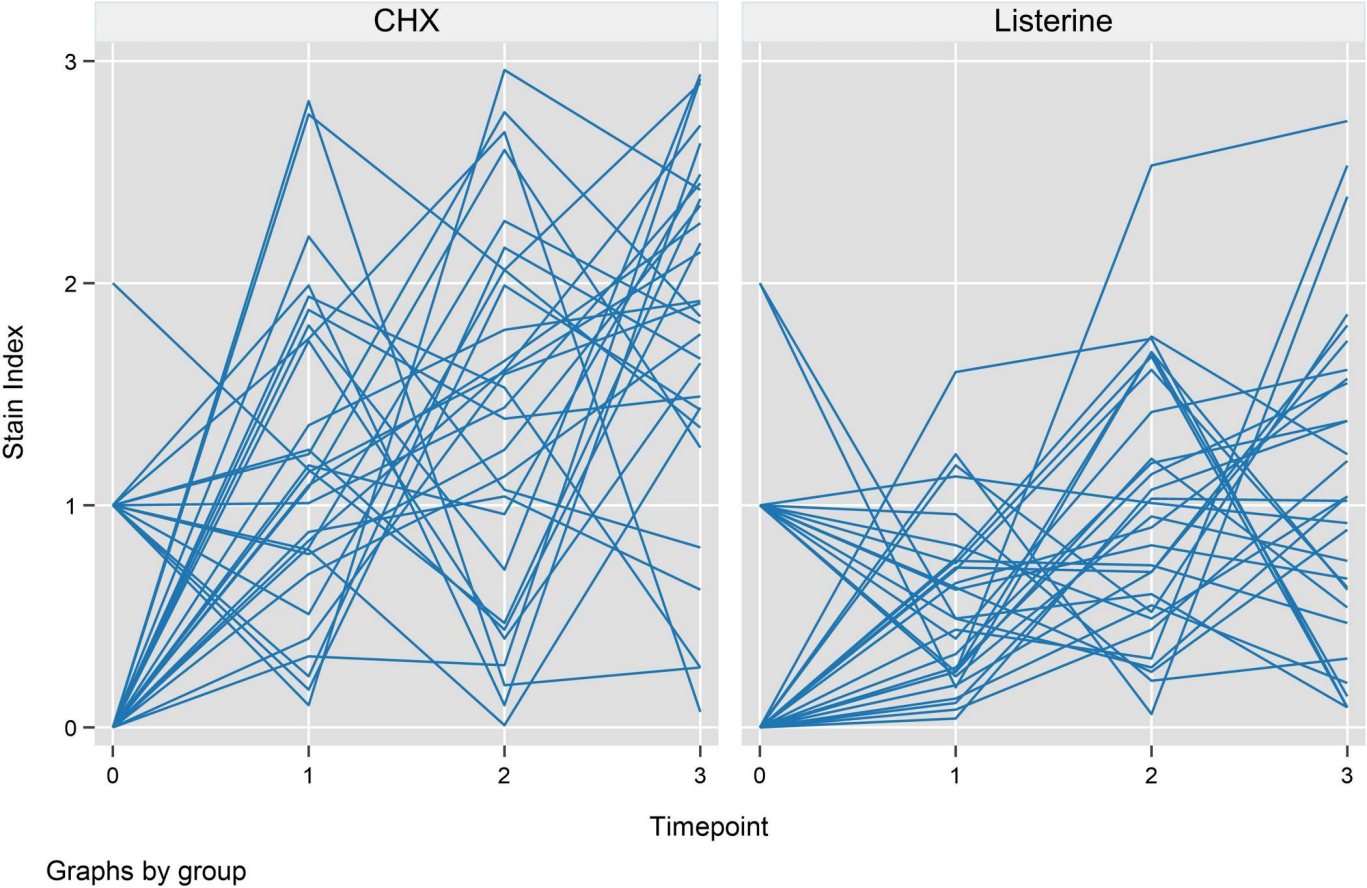
Individual plots per time point per treatment arm for staining.

### 3.6 Numbers Analyzed for Each Outcome, Estimation, and Precision, Subgroup Analyses

Population‐averaged GEE models with robust standard errors and an exchangeable correlation structure were fitted for the gingival index, the plaque index, and the stain index over time, adjusting for age and gender. For all outcomes, the main effects (except for gingival index treatment) and treatment time interactions were significant. Because the treatment × time interaction was significant for all outcomes, interpretation of the coefficients is not straightforward. Time coefficients represent differences from baseline time for the CHX group (baseline group), and the coefficients for treatment show the differences between treatment arms at baseline. Therefore, it is easier to assess the Wald tests and interpret the results using the marginal plots (Figure 6) that show differences with elements of precision (95% CIs) between arms over time.

**Figure 6:**
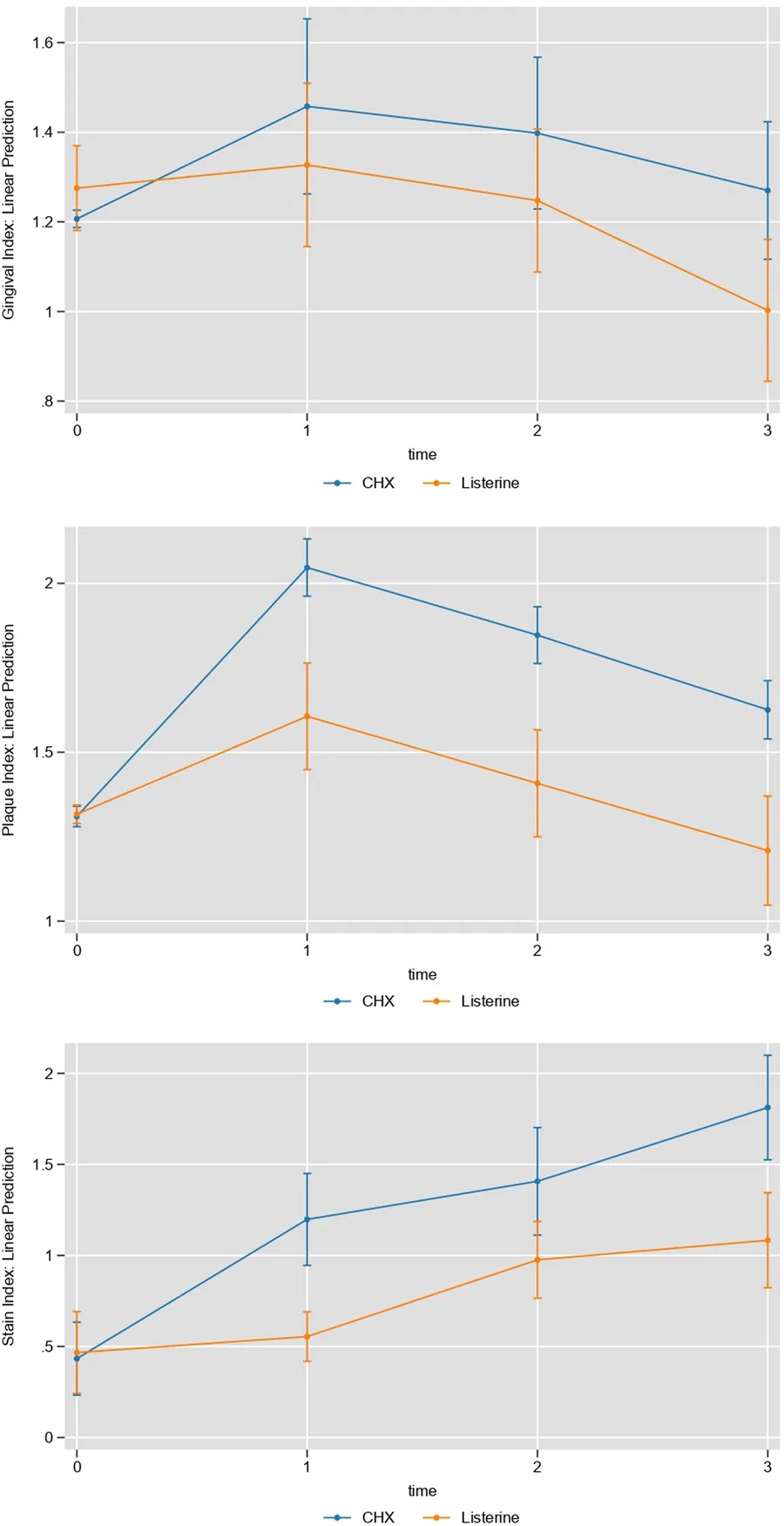
Model‐estimated marginal mean values with 95% confidence intervals for gingival index, plaque index, and stain index over time in the chlorhexidine and essential oil mouthrinse groups, based on the fitted generalized estimating equation models.

The Wald test and model‐estimated marginal means presented in Figure 6 showed the following:

GI: no overall difference between arms (*p* = 0.07), but significant change over time (*p* = 0.02). The significant interaction (*p* = 0.02) indicates that the evolution of the outcome differed between arms over time.

PI: Significant difference between arms (*p* < 0.001), with higher PI scores for CHX compared to EOM, significant change over time (*p* < 0.001, and the evolution of the outcome differed between arms over time (*p* < 0.001),

Stain: Significant difference between arms (*p* < 0.001), with greater staining in the CHX group compared to EOM, significant change over time (*p* < 0.001); also, the evolution of the outcome differed between arms over time (*p* < 0.01) (Table 3).

**Table 3:** Estimates, 95% confidence intervals, and *p*‐values derived from generalized estimating equation (GEE) models for the effect of mouthwash over time adjusted for age and gender on the gingival index, the plaque index, and the stain index.

Outcome	Predictor	Category	Estimate (*β*)	95% CI	*p* value	Wald test *p* value
Gingival index	Intervention	CHX	Reference			0.07
		EOM	0.08	−0.02to 0.18	0.12	
	Follow‐up time	Baseline	Reference			0.02
		1 month	0.25	0.06 to 0.45	0.01	
		2 months	0.19	0.02 to 0.36	0.03	
		3 months	0.06	−0.09 to 0.22	0.42	
	Group × time	EOM × 1 month	−0.20	−0.48 to 0.09	0.17	0.02
		EOM × 2 months	−0.22	−0.48 to 0.04	0.10	
		EOM × 3 months	−0.34	−0.57 to −0.10	0.01	
	Age	Per year	−0.003	−0.01 to 0.003	0.27	
	Gender	Female	Reference			
		Male	0.08	−0.04 to 0.19	0.19	
Plaque index	Intervention	CHX	Reference			< 0.001
		EOM	0.02	−0.04 to 0.07	0.53	
	Follow‐up time	Baseline	Reference			< 0.001
		1 month	0.74	0.64 to 0.84	< 0.001	
		2 months	0.534	0.44 to 0.63	< 0.001	
		3 months	0.32	0.22 to 0.42	< 0.001	
	Group × Time	EOM × 1 month	−0.45	−0.63 to −0.26	< 0.001	< 0.001
		EOM × 2 months	−0.45	−0.630 to −0.26	< 0.001	
		EOM × 3 months	−0.42	−0.61 to −0.24	< 0.001	
	Age	Per year	−0.002	−0.01 to 0.01	0.64	
	Gender	Female	Reference			
		Male	0.12	−0.02 to 0.25	0.08	
Stain index	Intervention	CHX	Reference	Reference	Reference	< 0.001
		EOM	0.03	−0.28 to 0.33	0.87	
	Follow‐up time	Baseline	Reference			< 0.001
		1 month	0.77	0.40 to 1.13	< 0.001	
		2 months	0.97	0.62 to 1.33	< 0.001	
		3 months	1.38	1.06 to 1.69	< 0.001	
	Group × Time	EOM × 1 month	−0.68	−1.13 to −0.22	0.003	< 0.01
		EOM × 2 months	−0.47	−0.94 to 0.01	0.05	
		EOM × 3 months	−0.76	−1.28 to −0.24	0.004	
	Age	Per year	0.004	−0.004 to 0.01	0.37	
	Gender	Female	Reference			
		Male	0.02	−0.10 to 0.14	0.70	

### 3.7 Harms

No adverse events or harmful effects were reported during the study period. None of the participants experienced oral irritation, burning sensation, allergic reactions, or any other clinically significant side effects related to the use of either mouthwash. No participant discontinued the intervention due to any adverse effect.

## 4 Discussion

### 4.1 Main Findings in the Context of the Existing Evidence, Interpretation

Our results showed that both mouthwashes produced changes in the plaque index, the gingival index, and the stain index over time. The gingival index decreased in both groups during the follow‐up period, and although the pattern of reduction differed between groups over time, no overall difference between the two mouthrinses was observed. The EOM group consistently showed lower plaque index scores from the first follow‐up time point onward compared to the CHX group. More staining was observed in the CHX group from the first follow‐up visit onward.

The comparative interpretation of the plaque index findings should consider the oral hygiene protocol used in the present study. Participants were instructed to use the assigned mouthwash immediately after routine toothbrushing, and no standardized waiting interval was prescribed. Because certain toothpaste constituents may reduce chlorhexidine substantivity, this protocol may have attenuated the clinical effect of CHX and could therefore have contributed to the observed difference in the plaque index between the groups. Accordingly, the significantly lower plaque index observed with the EOM should be interpreted keeping this potential limitation in mind. In addition, although allocation concealment and outcome‐assessor blinding were maintained, complete participant blinding could not be guaranteed because the two mouthwashes differed in taste and aroma, and their bottle shapes differed slightly. Formal assessment of blinding success was not performed, and differential treatment perception may therefore have influenced adherence or oral hygiene behavior.

The use of a wide spectrum of antimicrobial mouthrinses in addition to mechanical methods has been reported to improve the maintenance of an adequate control of periodontal health (Zaitouna et al. 2017). Although the effects and side effects of antiseptic agents have been extensively investigated in general dental practice, the potential role of antimicrobial mouthrinses as adjuncts to usual oral hygiene methods in orthodontic patients has not been adequately explored (Alshehri 2018). However, it has been widely demonstrated that fixed appliances increase biofilm accumulation, as orthodontic brackets create additional plaque‐retentive sites that make proper oral hygiene more difficult and consequently affect periodontal health, with a higher risk for gingivitis and periodontitis (Weber et al. 2023). Therefore, the present study compared the effectiveness of the two main chemotherapeutic agents used in mouthwashes, CHX and essential oils, to assess their potential effects on the periodontal health of orthodontic patients undergoing fixed appliance therapy over a 3‐month period.

Our findings are partially consistent with evidence from previous studies conducted in non‐orthodontic populations. In a 6‐month controlled clinical trial, Zaitouna et al. (2017) also reported that the EO and CHX mouthwashes achieved a comparable reduction in plaque and gingival indices in the long term. Additionally, although both agents induced a higher risk of stain development compared to controls, the level of stains in the CHX group was significantly greater than that in the EO group.

In a systematic review and meta‐analysis by Van Hussain et al. (2025) comparing the effects of CHX and EO mouthwashes, CHX was reported to significantly reduce plaque retention compared to EO during long‐term use (more than 1 month). However, according to the present findings, gingival outcomes were similar between groups, whereas CHX use was associated with significantly higher stain formation. They included two studies in the meta‐analysis, and the weighted mean difference after 1 month of follow‐up was very small and not clinically relevant (WMD = 0.46; 95% CI: 0.09 to 0.84; *p* = 0.01). Therefore, these results are almost similar to the current study.

Using an in situ model to evaluate the antiplaque effect after 4 days of using EO and CHX mouthwashes, Singh et al. (2013) reported that EO and 0.2% chlorhexidine showed a high antiplaque effect compared to controls. However, although 0.2% chlorhexidine produced a greater reduction of the thickness and surface covering grade by the biofilm, both antiseptics showed high and similar antibacterial activity, suggesting that EO could be considered a reliable alternative to CHX and that their daily use may be effective in the reduction of dental plaque formation in the short term.

The scientific evidence on the efficacy of different mouthrinses in orthodontic patients is still lacking, especially considering the use of EO. A meta‐analysis by Hussain et al. (2023) showed that adjunctive use of CHX in orthodontic patients undergoing fixed therapy is associated with improved gingival inflammation and plaque control compared with mechanical cleaning alone, but, in the long term, potential adverse effects induced by CHX, such as staining teeth, taste alteration, or oral mucosa irritation, should be taken into account, especially in children (Van Leeuwen et al. 2011).

In a randomized clinical trial, Raju et al. (2017) compared the effectiveness of three agents (CHX, EO, and green tea) in mouthwashes as adjuncts to toothbrushing for gaining some benefits in oral hygiene and maintenance after 15 days. All three mouthwashes were found to be effective against plaque accumulation and gingivitis. However, when compared across different groups, although green tea mouthwash showed the highest effectiveness in maintaining periodontal health, EOM appeared to be more efficient in the reduction of plaque and gingival indices compared to CHX mouthrinses.

In accordance with the present results, the better performance of EOM could be explained by considering the different mechanisms of action between CHX and EO. Fixed orthodontic appliances create a distinct oral environment that favors the development of a different type of dental biofilm. Brackets, bands, and archwires increase plaque‐retentive sites and promote the formation of thicker, more mature, and more complex biofilms. This orthodontics‐associated biofilm is typically more anaerobic, rich in extracellular matrix, and more difficult to disrupt than plaque formed on smooth tooth surfaces. Essential oil (EO) molecules, due to their lipophilic nature, are able to penetrate the hydrophobic regions of mature biofilms more effectively, whereas chlorhexidine (CHX) primarily exerts its action by binding to surface components (Van Strydonck et al. 2012; Whitaker et al. 2000). Although CHX shows high substantivity through strong binding to the dental pellicle, enamel, and soft tissues, its effectiveness may be reduced in the presence of fixed orthodontic appliances. Metallic and composite components can interfere with CHX adsorption, and plaque tends to accumulate in protected niches around brackets and wires, where diffusion of CHX is limited. Consequently, despite its strong antibacterial properties, the practical antiplaque effect of CHX around orthodontic appliances may be diminished (Ouhayoun 2003).

Moreover, the use of a CHX mouthwash has also been associated with a significant shift in the microbiome toward more anaerobic bacteria, leading to more acidic conditions and lower nitrite availability in healthy individuals as early as 7 days (Gkinosati et al. 2025). These microbial changes may increase the risk of oral disease, as lower microbial diversity after using CHX has been related to greater risk of periodontal diseases. Therefore, the negative effect of CHX on oral nitrite synthesis is another important aspect to consider, as nitrite has been shown to inhibit the growth of periodontal bacteria and to reduce acid production by these microorganisms (Gkinosati et al. 2025). Additionally, it should be noted that limited patient compliance is also reported in the long‐term use of CHX due to its adverse effects, mainly an increased level of formation of extrinsic tooth stain and calculus deposition compared to EO (Zaitouna et al. 2017).

The effectiveness of EO mouthrinses has been extensively described in the literature, especially when used as an adjunct to usual oral hygiene procedures in the long‐term period (Bescos et al. 2020; Sharma et al. 2004; Fornell et al. 1975) Additionally, similar to the present results, several previous studies with more than 1 month of follow‐up have reported that EO represents a reliable alternative to chlorhexidine mouthwash with respect to parameters of gingival inflammation, while significantly reducing the formation of extrinsic tooth stains (Hussain et al. 2025). However, in the present study, a greater antiplaque action was also associated with the use of EO compared to the CHX group.

### 4.2 Limitations

The study has several limitations. First, it was conducted at a single center in a relatively selected orthodontic population, limiting generalizability to other populations and clinical settings. Exclusion of individuals with systemic conditions, recent periodontal treatment, tobacco use, allergies to study ingredients, or additional oral hygiene interventions may further limit applicability to patients with more complex medical histories or different oral hygiene behaviors. Second, the 3‐month follow‐up was insufficient to assess the long‐term comparative effectiveness, tolerability, staining, or other adverse effects of chlorhexidine and essential oil mouthwashes. Longer follow‐up is needed to determine whether the observed effects persist throughout fixed orthodontic treatment. Third, adherence was assessed through self‐reporting and visual estimation of remaining mouthwash at monthly visits. Returned volumes were not quantitatively measured, missed doses were not formally documented, and no predefined adherence threshold was used; therefore, actual twice‐daily adherence could not be objectively verified, and between‐group differences in adherence may have introduced measurement error and affected treatment effects. Fourth, although outcome‐assessor blinding and allocation concealment were maintained, complete participant blinding could not be guaranteed because of differences in taste, aroma, and slight bottle‐shape differences, and blinding success was not formally assessed. Treatment perceptions may therefore have influenced adherence, oral hygiene behavior, or subjective reporting. Fifth, no standardized interval between toothbrushing and mouthwash use was prescribed. Because some toothpaste constituents may interfere with chlorhexidine substantivity, immediate post‐brushing use may have attenuated its effect and should be considered when interpreting plaque index differences and comparing findings with studies using standardized intervals. Sixth, microbiological analysis of plaque or biofilm composition was not performed; therefore, the study provides clinical rather than microbiological evidence, and the mechanisms underlying the observed differences remain uncertain. Seventh, both adolescents and adults were included. Although only 9/60 participants (15%) were younger than 18 years of age, age heterogeneity may have introduced biological variation in gingival responses, particularly due to pubertal hormonal influences. Age was prespecified and adjusted for in the GEE models, but residual confounding cannot be excluded, and the small number of participants younger than 18 years of age prevented meaningful age‐stratified analyses. Finally, no placebo or no‐mouthwash control group was included. Thus, the study primarily assesses the comparative effects of chlorhexidine and essential oil mouthwash rather than their independent effects beyond routine mechanical oral hygiene. Future multicenter studies with longer follow‐up, objective adherence measurement, standardized mouthwash timing, formal blinding assessment, and microbiological outcomes are warranted.

### 4.3 Generalizability

The generalizability of these results might be limited because this study was conducted at a single center.

## 5 Conclusions

Both mouthwashes were associated with improvements in periodontal health over the 3‐month follow‐up. Compared with chlorhexidine, the EOM group demonstrated significantly lower plaque index and stain index scores, whereas no statistically significant overall difference between groups was observed for the gingival index, despite differences in its trajectory over time.

## Author Contributions

**Fazli Rabi:** conceptualization (equal), data collection (equal), investigation, writing – review and editing (equal), final approval. **Naveed Sadiq:** conceptualization (equal), Data curation (equal), supervision, methodology (equal), writing – review and editing (equal), final approval. **Muhammad Jamil:** conceptualization (equal), data entry (equal), writing – review and editing (equal), final approval. **Sadia Asif Samdani:** conceptualization (equal), literature review (equal), methodology (equal), writing – review and editing (equal), final approval. **Waqas Naseem:** conceptualization (equal), design, data analysis (equal), writing manuscript (equal), methodology (equal), writing – review and editing (equal), final approval. **Ferdous Bukhary:** design (equal), literature review (equal), methodology (equal), writing – review and editing (equal), final approval. **Umar Hussain:** conceptualization (equal), design, data analysis (equal), writing manuscript (equal), methodology (equal), writing – review and editing (equal), final approval. **Alessandra Campobasso:** Design (equal), literature review (equal), methodology (equal), writing – review and editing (equal), final approval. **Nikolaos Pandis:** data analysis (equal), data curation (equal), data interpretation (equal), writing – review and editing (equal), final approval.

## Funding

This research did not receive any specific grant from funding agencies in the public, commercial, or not‐for‐profit sectors. The study was self‐funded by the authors.

## Consent

The authors have nothing to report.

## Conflicts of Interest

The authors declare no conflicts of interest.

## Data Availability Statement

The data that support the findings of this study are available on request from the corresponding author. The data are not publicly available due to privacy or ethical restrictions. Data of the current study are available from the corresponding author on reasonable request. However, for privacy reasons, no individual data allowing identification of participants (e.g., videos) can be provided.

## Supporting information


**Figure S1:** Average stain index per time point and spaghetti individual plots with median splines per time point per treatment arm.
