# Supplementary material for: Comparison of Clinical Effects of Essential Oil–Containing Mouthwash and Chlorhexidine Mouth Wash in Patients Undergoing Fixed Orthodontic Treatment – Three‐Month Follow‐up Randomized‐Controlled Trial
Source: Clin Exp Dent Res. 2026 Oct 6;12(5):e70477. doi: 10.1002/cre2.70477 (PMC13640973; doi:10.1002/cre2.70477)
Supplement: Supplementary file 1 [file CRE2-12-e70477-s001.docx]

**Supplemental file with manuscript**

Fig S1. Average stain index per time point and spaghetti individual plots with median splines per time point per treatment arm.

**References**

1.	Kado I, Hisatsune J, Tsuruda K, Tanimoto K, Sugai M. The impact of fixed orthodontic appliances on oral microbiome dynamics in Japanese patients. Scientific Reports. 2020;10(1):21989.

2.	Zhao M, Yu C, Su C, Wang H, Wang X, Weir MD, et al. Dynamic effects of fixed orthodontic treatment on oral health and oral microbiota: a prospective study. BMC Oral Health. 2024;24(1):1537.

3.	Di Spirito F, Amato A, Di Palo MP, Cannatà D, Giordano F, D’Ambrosio F, et al. Periodontal management in periodontally healthy orthodontic patients with fixed appliances: an umbrella review of self-care instructions and evidence-based recommendations. Dentistry Journal. 2023;11(2):35.

4.	Hussain U, Alam S, Rehman K, Antonoglou GN, Papageorgiou SN. Effects of chlorhexidine use on periodontal health during fixed appliance orthodontic treatment: a systematic review and meta-analysis. European Journal of Orthodontics. 2023;45(1):103-14.

5.	Brookes ZL, Belfield LA, Ashworth A, Casas-Agustench P, Raja M, Pollard AJ, et al. Effects of chlorhexidine mouthwash on the oral microbiome. Journal of Dentistry. 2021;113:103768.

6.	Alavi S, Yaraghi N. The effect of fluoride varnish and chlorhexidine gel on white spots and gingival and plaque indices in fixed orthodontic patients: A placebo-controlled study. Dental research journal. 2018;15(4):276-82.

7.	Raju R, Divya A, Rajendran G, John JR, John J. Analogous assay between green tea mouthwash, listerine mouthwash and chlorhexidine mouthwash in plaque reduction, on orthodontic patients: a randomized cross-over study. Inter J Community Med Public Health. 2017;4(5):1429-35.

8.	Charles C, Mostler K, Bartels L, Mankodi S. Comparative antiplaque and antigingivitis effectiveness of a chlorhexidine and an essential oil mouthrinse: 6‐month clinical trial. Journal of Clinical Periodontology. 2004;31(10):878-84.

9.	Singh A, Daing A, Dixit J. The effect of herbal, essential oil and chlorhexidine mouthrinse on de novo plaque formation. International journal of dental hygiene. 2013;11(1):48-52.

10.	Quintas V, Prada-Lopez I, Donos N, Suarez-Quintanilla D, Tomas I. Antiplaque effect of essential oils and 0.2% chlorhexidine on an in situ model of oral biofilm growth: a randomised clinical trial. PLoS One. 2015;10(2):e0117177.

11.	Yaneva BK, Dermendzhieva YB, Mutafchieva MZ, Stamenov NV, Kavlakova LB, Tanev MZ, et al. Randomised controlled trial comparing the clinical effectiveness of mouthwashes based on essential oils, chlorhexidine, hydrogen peroxide and prebiotic in gingivitis treatment. Folia Medica. 2022;64(4):588-95.

12.	Kouadio A-A, Struillou X, Bories C, Bouler J-M, Badran Z, Soueidan A. An in vitro analysis model for investigating the staining effect of various chlorhexidine-based mouthwashes. Journal of Clinical and Experimental Dentistry. 2017;9(3):e410.

13.	Goes P, Dutra CS, Lisboa MR, Gondim DV, Leitão R, Brito GA, et al. Clinical efficacy of a 1% Matricaria chamomile L. mouthwash and 0.12% chlorhexidine for gingivitis control in patients undergoing orthodontic treatment with fixed appliances. Journal of oral science. 2016;58(4):569-74.

14.	Alshehri FA. The use of mouthwash containing essential oils (LISTERINE®) to improve oral health: A systematic review. The Saudi dental journal. 2018;30(1):2-6.

15.	Weber J, Bonn EL, Auer DL, Kirschneck C, Buchalla W, Scholz KJ, et al. Preprocedural mouthwashes for infection control in dentistry—an update. Clinical oral investigations. 2023;27(Suppl 1):33-44.

16.	Hussain U, Wahab A, Kamran MA, Alnazeh AA, Almoammar S, Alshahrani SSM, et al. Prevalence, Incidence and Risk Factors of White Spot Lesions Associated With Orthodontic Treatment–A Systematic Review and Meta‐Analysis. Orthodontics & Craniofacial Research. 2025;28(2):379-99.

17.	Van Leeuwen M, Slot D, Van der Weijden G. Essential oils compared to chlorhexidine with respect to plaque and parameters of gingival inflammation: a systematic review. Journal of Periodontology. 2011;82(2):174-94.

18.	Van Strydonck DA, Slot DE, Van der Velden U, Van der Weijden F. Effect of a chlorhexidine mouthrinse on plaque, gingival inflammation and staining in gingivitis patients: a systematic review. Journal of Clinical Periodontology. 2012;39(11):1042-55.

19.	Whitaker E, Pham K, Feik D, Rams T, Barnett M, Pan P. Effect of an essential oil‐containing antiseptic mouthrinse on induction of platelet aggregation by oral bacteria in vitro. Journal of clinical periodontology. 2000;27(5):370-3.

20.	Ouhayoun JP. Penetrating the plaque biofilm: impact of essential oil mouthwash. Journal of Clinical Periodontology. 2003;30:10-2.

21.	Gkinosati AA, Makrygiannakis MA, Kaklamanos EG. Effects of mouthwashes on the morphology, structure, and mechanical properties of orthodontic materials: a systematic review of randomized clinical studies. European Journal of Orthodontics. 2025;47(4):cjaf048.

22.	Bescos R, Ashworth A, Cutler C, Brookes ZL, Belfield L, Rodiles A, et al. Effects of Chlorhexidine mouthwash on the oral microbiome. Scientific Reports. 2020;10(1):5254.

23.	Fornell J, Sundin Y, Lindhe J. Effect of Listerine® on dental plaque and gingivitis. European Journal of Oral Sciences. 1975;83(1):18-25.

24.	Sharma N, Charles C, Lynch M, Qaqish J, McGuire J, Galustians J, et al. Adjunctive benefit of an essential oil–containing mouthrinse in reducing plaque and gingivitis in patients who brush and floss regularly: a six-month study. The Journal of the American Dental Association. 2004;135(4):496-504.
